# Treatment of Syphilis

**Published:** 1888-02

**Authors:** 


### *TREATMENT OF SYPHILIS.*

In the issue of the *Bulletin Gén. de Thérapeutique* of October 30th, we find a useful paper on the treatment of syphilis, by Prof. Verneuil. As a representative of the more conservative of French surgeons, Verneuil speaks with authority on such topics. The conclusions at which he arrives harmonize with the opinions most generally held.

He maintains the superiority of mercury. As respects the diagnostic value of the two agents—iodides and mercury—he never decides the question of specific lesion or not, except from the results of a trial of mercury. In three examples of old syphiloma of the testicle—cited for illustration—the iodide of potassium in massive doses failed to disperse the tumor, but mercurial treatment effected a cure in a few weeks, thus demonstrating the nature of the neoplasm.

Professor Verneuil does not advocate the huge doses of iodide of potassium now in vogue—2 to 3 grammes (30 to 45 grains) per day being his maximum—except in cases of rapidly destructive ulcerations of the nares, veil of the palate, and similar lesions, and even then in quantity not exceeding 75 or 96 grains *per diem.* He has never favored the conjoint administration of mercury and iodides. He prefers to give mercury by itself, and associated with remedies to improve the general state of the patient. He has occasionally made use of the combination of these remedies in slowly developing secondary or tertiary accidents when mercury does not act well, or has not been given at all. Under such circumstances, he prescribes in the simplest way ¾ grain of protoiodide of mercury and 15.5 grains of potassium iodide.

Mercurial frictions, although, in some cases, acting energetically, do not commend themselves to his judgment. When he has employed inunction, he has not dispensed with the internal administration of the protoiodide or some other mercurial, in small doses. Nor has he practiced the method of subcutaneous injection of mercurials, which often cures, apparently, in twenty to thirty days. He holds that the most certain curative results are obtained by the slow saturation of the organism as effected by the stomachal administration, rather than by sudden impression.

For the local treatment of syphilitic ulcerations, mucous patches, etc., the early manifestations of the constitutional state, he employs nitrate of silver, or chloral solutions, topically, in conjunction with the use of mercury internally.

